# The Online Education Mode and Reopening Plans for Chinese Schools During the COVID-19 Pandemic: A Mini Review

**DOI:** 10.3389/fpubh.2020.566316

**Published:** 2020-12-10

**Authors:** Xuanzhen Cen, Dong Sun, Ming Rong, Gusztáv Fekete, Julien S. Baker, Yang Song, Yaodong Gu

**Affiliations:** ^1^Faculty of Sports Science, Ningbo University, Ningbo, China; ^2^Savaria Institute of Technology, Eötvös Loránd University, Szombathely, Hungary; ^3^Department of Sport and Physical Education, Hong Kong Baptist University, Hong Kong, China; ^4^Faculty of Engineering, University of Szeged, Szeged, Hungary

**Keywords:** online education, back-to-school, corona virus disease, novel coronavirus, reopening time

## Abstract

Recently, an unprecedented coronavirus pandemic has emerged and has spread around the world. The novel coronavirus termed COVID-19 by the World Health Organization has posed a huge threat to human safety and social development. This mini review aimed to summarize the online education mode and plans for schools to resume full-time campus study in China during COVID-19. Chinese schools have made significant contributions to the prevention and control of the transmission of COVID-19 by adopting online learning from home. However, normal opening and classroom teaching have been affected. For education systems at all levels, online education may be an effective way to make up for the lack of classroom teaching during the epidemic. To protect staff and students from COVID-19, the timing of students returning to full-time campus study needs to be considered carefully. Reviewing and summarizing of the Chinese education system's response to the virus would be of great value not only in developing educational policy but also in guiding other countries to formulate educational countermeasures.

## Introduction

Since December 2019, atypical pneumonia has been widely spreading throughout the world (1), and the number of recorded cases has increased dramatically. On February 11, 2020, the International Committee on Taxonomy of Viruses (ICTV) announced that the new virus responsible for atypical pneumonia would be named “severe acute respiratory syndrome coronavirus 2 (SARS-CoV-2).” The name created unintended fear in the public, especially in Asia which was affected badly by SARS in 2003. From a risk communications perspective, the World Health Organization (WHO) has begun announcing “the COVID-19 virus” as the name of the new virus when communicating and providing updates for the public (2). This new outbreak has impacted massively on worldwide populations, especially on health and economy toll globally (3). The outbreak of COVID-19 has also resulted in huge health and economical toll globally. Modeling and analysis have revealed that even if the outbreak is effectively controlled, the global economy will experience a massive decline (4). In China, for example, COVID-19 spread rapidly from a single city to the whole country in just 30 days since the outbreak (5). This rate of transmission is alarming, and the transmission seems to be taking place via individuals in close contact. Studies have shown that COVID-19 has a higher susceptible population, is more widespread, and is more contagious than the SARS coronavirus (6). As of May 26, 2020, the total number of confirmed cases in China reached 84,543. Worldwide, the number of recorded cases has reached 5,505,801 (7).

It should be mentioned, that the outbreak coincided with the traditional Spring Festival, which is the most important festival of the year in China. During this time, several hundred million people move and relocate, and residents and tourists mostly choose to travel by using crowded planes, trains, and buses (8). This means that infected individuals may have close contact with other individuals for long periods when traveling long distances (9), which leads that the spread of COVID-19 was hardly controlled. The rapid spread of the epidemic which is due in part to large population movements is considered one of the main reasons for the large-scale transmission of the disease (9, 10). Fortunately, schools were closed during the Chinese Lunar New year that enabled the government to carefully consider measures needed for schools to protect staff and students. China has a large and dense student population located in both urban and rural areas. According to statistics recorded in 2018 (11), there are 518.8 thousand schools of all levels and types in China, with 276 million students and 16.7 million full-time teachers. Social isolation is undoubtedly effective in reducing virus transmission, although COVID-19 rarely affects children in asymptomatic cases, and infection and virus transmission characteristics are not clear in this group (12).

At present, the most important method of containment during the COVID-19 Pandemic is isolation (13). As the most severely affected area in China, Wuhan city was in lockdown on January 23, 2020. The subsequent spread of the COVID-19 virus led to the imposition of a cordon sanitaire, limiting the population movements among 16 cities in Hubei province and affecting more than 50 million people (13). And this restriction has gradually expanded to most areas of China. Public transport including planes, high-speed trains and subways, entertainment venues including cinemas, bars and amusement parks, and schools at all levels were forced to close, leading to an unprecedented nationwide lockdown in China (8, 13). Despite quarantine representing an effective method of curbing viral spread, WHO advises against the enforcement of limitations to travel and trade (13, 14).

In response to the COVID-19 epidemic, the Chinese educational mode is transiting online. During the outbreak, the Ministry of Education of the People's Republic of China (MOE) launched an emergency policy of Suspending Classes without Learning Termination (SCWLT) to encourage the development of online education and resource sharing (15). Online education has partly made up for the lack of classroom teaching provision during the crisis. However, deficiencies in network environments and hardware facilities are still worthy of attention. Another challenge is making a new plan for the reopening of schools. Closing schools and postponing the reopening of schools are a mitigation measure during the current COVID-19 epidemic. Although these measures are conducive to reducing the spread of the virus, they cannot be advocated as a long-term solution.

The main purpose of this paper was to summarize the online education mode and plans for schools to resume a full-time campus study adopted by the Chinese education system at all levels during the outbreak of COVID-19. An understanding and evaluation of the current condition of the Chinese education system would be of great value not only in creating further educational policies but also assisting in guiding other countries to formulate educational provision countermeasures.

## Viral Transmission and Spread in China

**Figure 1:**
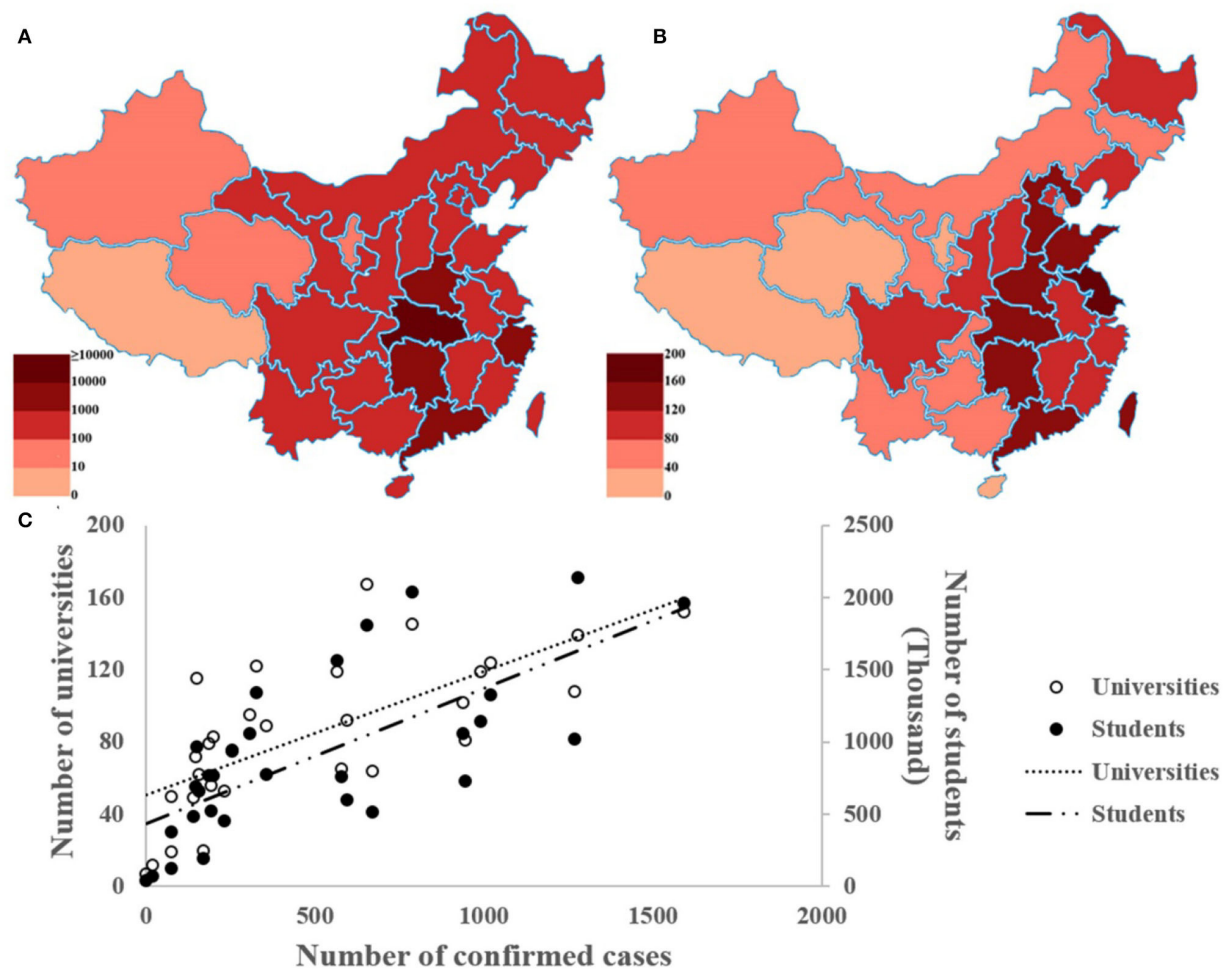
Correlation between the cumulative case and university distribution in China. **(A)** The cumulative number of confirmed cases in China. **(B)** The distribution of universities in China. **(C)** The Scatterplots of cumulative diagnosis and the number of universities and students in China. This figure is original and based on data from National Health Commission of the People's Republic of China (7) and Ministry of Education of the People's Republic of China (11).

As of May 26, 2020, 84,543 cases of COVID-19 were confirmed in China, 68,135 of which were confined to Hubei (7). Confirmed cases have been reported in 34 provinces, cities, and in the autonomous regions of China. Figure 1A outlines the distribution of cumulative confirmed cases in China (data as of May 26, 2020).

As the mainstay of Chinese society, Chinese universities have made important contributions to emergency risk management, including the collection of alumni resources, medical rescue and emergency management, mental health maintenance, personnel mobility control, and innovation in online education models (16). However, due to the large population base of the school (including students and staff), this also creates great challenges in the prevention of the disease and management of the school. China currently has 518,800 schools at all levels, including more than 2,600 universities (excluding Hong Kong, Macao, and the Taiwan regions) (11). If we consider the region of Hubei as an example, this province has 128 colleges and universities with a total enrollment of more than 1.438 million students. Most universities in the Hubei region are in Wuhan, the provincial capital, which is the most severely affected area. Figure 1B shows the distribution of colleges and universities in various provinces, cities, and autonomous regions across the country. Moreover, the cumulative number of cases, the number of universities, and the number of university students in each province are highly correlated (Figure 1C, excluding Hong Kong, Macao, and Taiwan regions) (11).

## Development of an Online Education Mode

Due to the outbreak of COVID-19, the normal teaching provision of all schools has been affected to varying degrees. During the extraordinary period of epidemic prevention and control, online education seems to make up for the lack of classroom teaching. Online education meets the needs of students and achieves high-quality resource sharing. Since the MOE issued the emergency policy of SCWLT in February 2020, efforts have been made to integrate high-quality teaching resources from the country, relevant provinces, cities, and schools to provide free learning resources during the delayed school term (17). Details of how the emergency policy will be implemented and what impacts it may cause are still being debated (18).

Online education can be traced back to correspondence education in the 19th century. Since the beginning of the 20th century, with the progress of communication technology, the way of learning has been constantly evolving, and the Internet and open-source learning have created an environment for large-scale distance education (18–20). There are more than 230,000 related companies engaged in the online education industry in China. In the past decade, online education companies have experienced explosive growth, and more than 60,000 related companies were further established in 2019. In the past 5 years, the concentration of online education increased, with more than 130,000 companies being established in 1–5 years, accounting for 57.6%.

Online education, through the application of information technology and Internet technology for content dissemination and distance learning, has to some extent reduced the risk of epidemic spread. The number of daily active users of education and learning apps during the epidemic period rose from 87 million to 127 million, an increase of 46%. Among the increase, the number of daily active users of basic education increased by more than 23 million (21). During the 8-week teaching period of the emergency policy of SCWLT, nearly 270 million students from universities, high schools, junior high schools, and primary schools in China were engaged in course learning online. At the same time, nearly 20 million teachers from universities, high school, junior high school, and primary schools performed teaching activities via the Internet. The SCWLT policy has created the largest number of online students and has refreshed the online currency of Internet use (15). Facing the big challenge of online education, whether it is a school, university, teacher, or parent, will take time to adapt to the process. Zhang et al. (18) summarized the difficulties in the implementation of the SCWLT. They suggested that there are at least five major difficulties in policy implementation that need to be addressed, including: (1) Infrastructure constraints; (2) Low proportion and low efficiency of the use of resources; (3) The outcome effect is contingent on teachers' teaching ability and experience; (4) A variety of unpredictable teaching and learning problems; (5) It remains unclear what pedagogical method may suit the online education best.

The traditional teaching method is “passive learning,” which is usually delivered in a large classroom, with little interaction. Teachers often occupy a dominant position in classroom teaching, which leads to the weakening of students' subjective innovation ability and critical spirit. Offline education mode generally has certain problems for students' including personalized learning and differentiated teaching. Online education, through the information database management technology and two-way interactive function of computer network, realizes the complete system tracking record of individual information and learning process, to provide a practical and effective way for personalized teaching. SCWLT is not a simple transition from classroom teaching/offline education to the Internet platform. Its essence is a large-scale and far-reaching practice of educational mode (15). Online education is a combination of teaching technology, education concepts, and traditional education to form a new educational mode suitable for modern educational requests. As 5G and artificial intelligence technologies develop, online education might effectively solve problems related to offline education. Although the key features of 5G Technology greatly increase the technology complexity, resulting in a much higher capital-intensive level compared with previous generations, most governments in developing countries tend to encourage 5G deployment as a strategic priority (22). Therefore, online and offline integration is imperative. At present, governments and schools are taking this opportunity and are encouraging teachers to test and validate online and offline teaching methods exploring this practice, using artificial intelligence, and big data to conduct academic analysis and educational evaluation to investigate, design, and implement future educational provision (15).

## Plans of the Reopening of Schools

**Figure 2:**
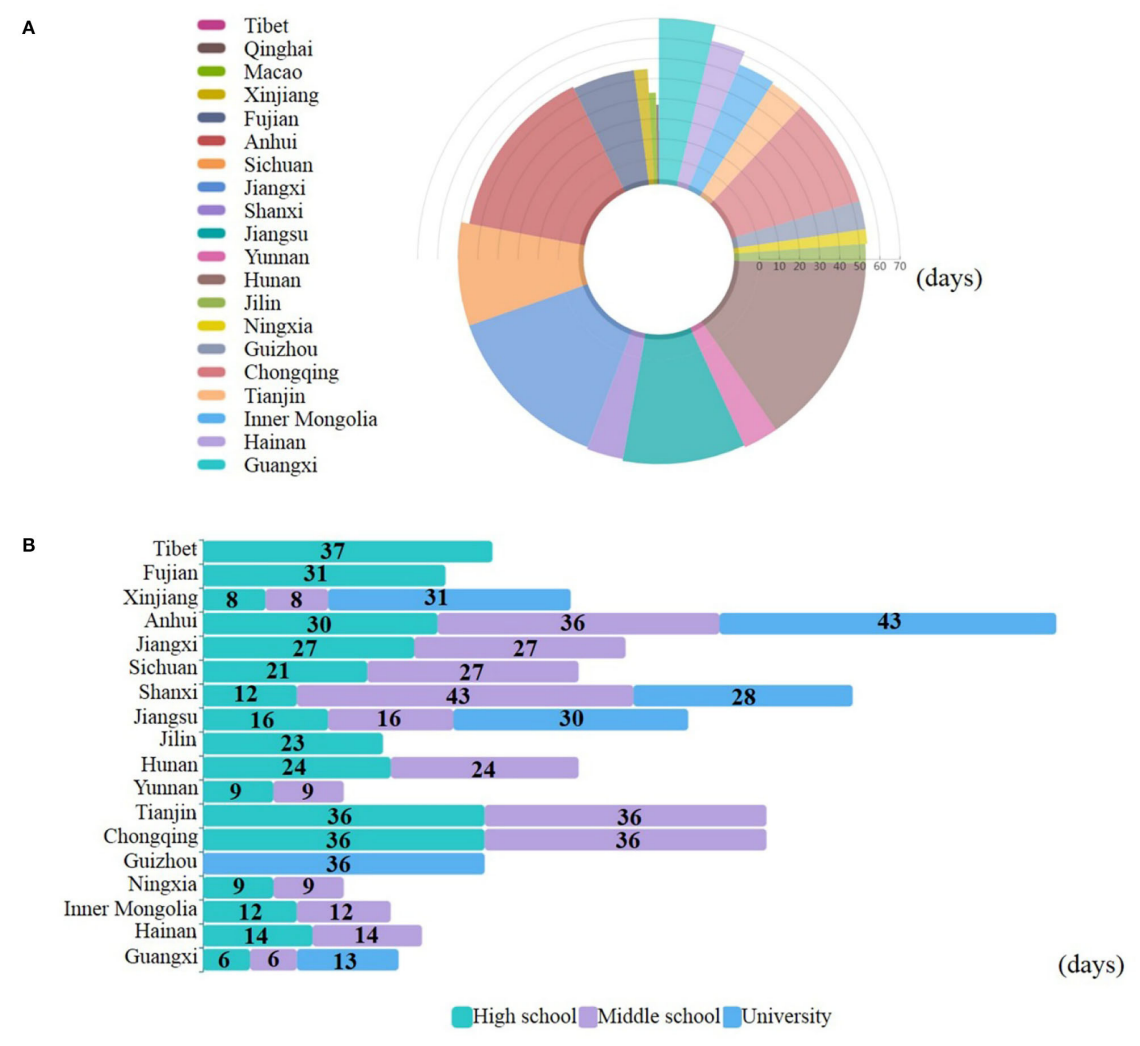
The status of the COVID-19 and the time of back-to-school in China's provinces. **(A)** The time from the first diagnosis to the first time of no existing case in each province. **(B)** The number of days between the first time of no existing case and the reopening times of high school, middle school, and university in each province. This figure is original and based on data from National Health Commission of the People's Republic of China (7).

As the transmission of the virus increased, students (including all ages and educational levels) in many regions of the world were forced to stay at home. This meant that all educational provision was affected. The 77th edition of the WHO's new outbreak report shows that 90% of students and more than 150 million children and young adults worldwide have been affected by school suspensions (23). In China, the epidemic situation has improved significantly since December 31, 2019, when atypical pneumonia in Wuhan was first reported. Figure 2A shows the number of days between the first diagnosis and the first time of no existing case in each province (data as of April 18, 2020). The number of confirmed cases in Tibet reached zero within 14 days of the first confirmed cases. It took Guangxi 70 days to achieve that goal. China's students are now starting to return to campus, and the new opening plan needs further careful consideration.

With the decreasing of infection rates across the country, most provinces and cities in China decided their own time of back-to-school at all levels based on the epidemiological evidence (Table 1). Figure 2B presents the number of days between the first time of existing cases of zero and the reopening times of high school, middle school, and university in each province (data as of April 18, 2020). In China, the back-to-school plan of each province was not made by the national central government, but by the local education management department, which was distributed the power by the central government. And the education management department of each province independently made the plan according to the epidemic situation. For most provinces, grade 3 students attending high and middle school are regarded as important objectives for back-to-school plans, as they are expected to progress to higher-level education. Another important group is university graduates, who are facing the pressure of employment. The decision making on the time of back-to-school based on the following five main points;

- The status of the COVID-19 epidemic in the province, including the cumulative cases, cumulative recovery, existing cases, presence of asymptomatic infections, etc.
- Different reopening plans depending on the school and grade level. Students in grade three of high schools and middle schools, as well as fresh university graduates, are generally preferred to return to education.
- The perfection and implementation of self-quarantine and disease prevention. The specific implementation includes self-monitored quarantine not <14 days after returning to school, health diagnosis of students from different areas, school disinfection work, etc.
- The connection between online courses and offline courses. Since students generally receive online education during school closure, they need to make a transition to offline education after returning to school. The educational experience should be of high quality and the quality of teaching should be ensured.
- The establishment of multiple collaborative governance mechanisms including “government-family-school-society.” The formulation of the back to school plan is not only realized by the school but also needs the resource and information sharing between the government, family, and society.

**Table 1:** The detailed information about the outbreak and the time of back-to-school in China's provinces.

**Provinces**	**Cumulative cases**	**Cumulative recovery**	**The time of the first case**	**The first time of no existing case**	**The time of back-to-school**		
					**Grade 3 of high school**	**Grade 3 of middle school**	**Universities**
Beijing	593	509	January 20	-	April 27	May 11	-
Tianjin	189	173	January 21	March 15	April 20	April 20	-
Hebei	328	316	January 22	-	April 23	May 7	-
Shanxi	197	135	January 22	March 13	March 25	April 25	April 10
Inner Mongolia	193	104	January 23	March 18	March 30	March 30	–
Liaoning	146	142	January 22	–	April 15	–	–
Jilin	102	97	January 22	March 15	April 7	–	–
Heilongjiang	892	472	January 22	–	April 7	April 13	–
Shanghai	628	512	January 20	–	April 27	April 27	–
Jiangsu	653	643	January 22	March 14	March 30	March 30	April 13
Zhejiang	1,268	1,246	January 21	–	April 13	April 13	April 26–May 10
Anhui	991	984	January 22	March 8	April 7	April 13	April 20
Fujian	355	336	January 22	March 7	April 7	–	–
Jiangxi	937	936	January 21	March 11	April 7	April 7	–
Shandong	787	765	January 21	–	April 15	–	–
Henan	1,276	1,254	January 21	–	April 7	–	–
Hubei	68,128	63,494	–	–		–	–
Hunan	1,019	1,015	January 21	March 14	April 7	April 7	–
Guangdong	1,579	1,482	January 21	–	April 27	April 27	–
Guangxi	254	252	January 22	April 1	April 7	April 7	April 14
Hainan	168	162	January 22	March 24	April 7	April 7	–
Chongqing	579	570	January 21	March 15	April 20	April 20	–
Sichuan	561	553	January 21	March 11	April 1	April 7	–
Guizhou	147	144	January 22	March 16	March 16	March 16	April 21
Yunnan	184	177	January 21	March 14	March 23	March 23	–
Tibet	1	1	January 29	February 12	March 20		–
Shanxi	256	252	January 23	–	March 30	April 7	–
Gansu	139	137	January 23	–	April 9	April 13	–
Qinghai	18	18	January 25	February 21	March 9–13	March 16–20	April 1–15
Ningxia	75	75	January 22	March 16	March 25	March 25	–
Xinjiang	76	73	January 23	March 8	March 16	March 16	April 8
Taiwan	398	178	January 30	–	–	–	–
Hong Kong	1,023	568	January 29	–	–	–	–
Macao	45	17	February 2	March 6	–	–	–
Total	84,185	77,792	–	–	–	–	–

*Data from National Health Commission of the People's Republic of China and as of April 18, 2020 (7); “-”: Data not presented or not applicable*.

Large population movements, particularly of college students from all over the country that are returning to school, and detection of possible transmission by asymptomatic carriers will also be a challenge to prevent and control further COVID-19 infections (24). The nation needs to be prepared for a possible rebound of the outbreak (5), especially in schools, with disastrous consequences and health implications that want to be avoided.

## Conclusions

Globally, the number of COVID-19 confirmed cases is still increasing. Although isolation alone may not be enough to prevent the spread of the epidemic, it is undoubtedly one of the most economical and effective ways to minimize its risk. For educational systems at all levels, online education may be an effective way to compensate for the lack of classroom teaching during the epidemic. However, issues such as the network environment, hardware equipment, and educational quality and educational experience for the students deserve special attention. From the perspective of the safety of teachers and students, whether it is primary, secondary school, or university, the organization of educational establishments reopening strategies need careful consideration.

## Author Contributions

XC, DS, and YS conceived the presented idea, developed the framework, and wrote the manuscript. MR, GF, JB, and YG provided critical feedback and contributed to the final version. All authors have read and agreed to the published version of the manuscript.

## Conflict of Interest

The authors declare that the research was conducted in the absence of any commercial or financial relationships that could be construed as a potential conflict of interest.
